# Diagnosis and Staging of Pediatric Non-Alcoholic Fatty Liver Disease: Is Classical Ultrasound the Answer?

**DOI:** 10.3390/pediatric13020039

**Published:** 2021-06-08

**Authors:** Angeliki Papachristodoulou, Dimitrios Kavvadas, Athanasios Karamitsos, Theodora Papamitsou, Maria Chatzidimitriou, Antonia Sioga

**Affiliations:** 1 Laboratory of Histology and Embryology, School of Medicine, Faculty of Health, Aristotle University of Thessaloniki, 541 24 Thessaloniki, Greece; apapachri@auth.gr (A.P.); kavvadas@auth.gr (D.K.); sioga@auth.gr (A.S.); 2 2nd Department of Ophthalmology, School of Medicine, Faculty of Health, Aristotle University of Thessaloniki, 541 24 Thessaloniki, Greece; thanoskaramitsos@auth.gr; 3 Department of Biomedical Sciences, School of Health Sciences, International University of Greece, 574 00 Thessaloniki, Greece; mchatzid952@gmail.com

**Keywords:** NAFLD, pediatric NAFLD/NASH, diagnosis, ultrasound, children, elastography, staging

## Abstract

The increased prevalence of non-alcoholic fatty liver disease (NAFLD) requires special attention in pediatric patients, as it manifests in them in a more severe and progressive way compared to adults. The implementation of the appropriate therapeutic interventions is determinant of the attempts to treat it. For that purpose, early diagnosis and staging of the disease is essential. The purpose of this review was to find and reveal the most appropriate diagnostic strategies and tools for diagnosis and staging of pediatric NAFLD/NASH based on their accuracy, safety and effectiveness. The methodology followed was that of the literature review. Particular emphasis was put on the recent bibliography. A comparative study of published articles about the diagnosis and management of pediatric NAFLD/NASH was also performed. In terms of diagnosis, the findings converged on the use of classical ultrasound. Ultrasound presented average sensitivity and specificity for diagnosing the disease in children, while in the adult population, sensitivity and specificity were significantly higher. Proton density fat fraction magnetic resonance imaging has been increasingly used for the diagnosis of steatosis in pediatric patients. Elastography is an effective tool for staging liver fibrosis and discriminating NASH from NAFLD in children. Even though liver biopsy is the gold standard, especially for NASH, it should be avoided for pediatric patients. Biochemical tests are less specific and less sensitive for the diagnosis of NAFLD, and some of them are of high cost. It seems that diagnostic imaging should be a first-line tool for the staging and monitoring pediatric NAFLD/NASH in order for appropriate interventions to be implanted in a timely way.

## 1. Introductory Elements

The rising prevalence of non-alcoholic fatty liver disease (NAFLD) and the possibility of its development into non-alcoholic steatohepatitis (NASH) and ultimately, to end-stage liver disease, makes its diagnosis a matter of great importance. Current management guidelines do not recommend screening tests for NAFLD in patients attending primary healthcare units or to high-risk patients (e.g., diabetic and obese patients). This is due to the unreliability of the diagnostic tools and to the lack of basic treatment options [1].

A meta-analysis reported that the risk for liver-induced mortality was 5.7 times higher for patients with NAFLD. Thus, it is suggested that the likelihood of NAFLD leading to morbidity and mortality depends on the severity of histological deterioration, especially in the presence of hepatic inflammation. Ref. [2] Children with pediatric NAFLD (pNAFLD) are at high risk of type 2 diabetes during early adulthood. Ref. [3] Recent data on the pediatric population have shown an alarming association among pNAFLD and cardiovascular risks, a finding that has not yet been confirmed for adults as well [4,5]. Progression of the disease could lead to the development of the severe fibrotic stage, which is referred to as pediatric non-alcoholic steatohepatitis (NASH) [6].

Given the aforementioned serious development of the disease in children, it is important to evaluate the diagnostic methods and tools in hand that are appropriate for this tender age.

## 2. Serological Markers and Diagnosis

The advantages of serological indicator analyses are their wide applicability, the reproducibility of the results and their high availability. However, none of them are completely specific to the liver, and the results can be affected by coexisting pathological conditions; therefore, meticulous evaluation is required. Ref. [7] CK18 (Cytokeratin 18, released by hepatocytes during necrosis or apoptosis) and soluble Fas factor and its ligand (Fas Ligand) (markers of the external apoptosis pathway) are the main indicators [8,9]. Screening for ALT (alanine aminotransferase) levels should be performed with caution, as there are baseline discriminations among ALT levels between healthy-weighted adults and children, apart from several other risk factors that could potentially affect them as well [10].

An alternative approach could be the recording of biomarkers related to oxidative stress and inflammation, produced by different oxidative pathways. In a study of histologically confirmed NASH patients, lipid peroxidation was systematically measured in a control group with similar characteristics (BMI, sex, age), and both oxidized LDL and thiobarbitic acid reactives were found to increase in NAFLD patients. Ref. [11] In a similar study using mass spectroscopy techniques, it was shown that plasma products of the free oxidative radicals of linoleic acid were significantly increased in adult patients with NASH compared with patients with NAFLD and individuals with normal findings from liver biopsies [12].

## 3. Severity of Hepatic Fibrosis, Histological Findings and Diagnosis

The stage of hepatic fibrosis is the most crucial prognostic indicator for NAFLD and predicts the risk of developing cirrhosis and complications. [13] Indeed, two new milestone studies have identified hepatic fibrosis as the strongest predictor of long-term NAFLD outcomes, including liver-related mortality and overall patient mortality [14,15].

NAFLD-associated hepatic fibrosis presents in different stages, ranging from the absence of fibrosis (stage F0) to cirrhosis (stage F4), with stages of fibrosis F2–F4 being considered clinically significant and stages F3–F4 being considered as advanced fibrosis. When interpreting non-invasive liver fibrosis tests, it is important to categorize the results based on a mild presence of fibrosis, a clinically significant fibrosis or an advanced fibrosis. Risk factors for the development of advanced fibrosis and cirrhosis are old age, severe obesity and the presence of metabolic syndrome [16]. Some routine clinical laboratory indicators have been created in recent years for the diagnosis and assessment of the severity of NAFLD. The most common is the AST (aspartate aminotransferase)/ALT (alanine aminotransferase) ratio (AST-to-ALT ratio (AAR [AST-to-ALT ratio])), while other indicators are the BARD score, the FIB4 index and the NAFLD fibrosis score (NFS) (in addition to the above, serum albumin values are included) [17].

Studies have shown that pediatric NAFLD exhibits features of NASH in almost half of the cases [18]. Pediatric NASH presents a unique histological pattern in comparison to adults [19]. There are two types of NASH proposed for describing the histologic evaluation: Type 1 is characterized by steatosis, ballooning degeneration and perisinusoidal fibrosis in both pediatric and adult patients, and Type 2, which is seen exclusively in pediatric patients, by the absence of ballooning degeneration and perisinusoidal fibrosis. Type 2 has mostly been observed in obese children of early age [18]. Those aforementioned characteristics make biopsy the “gold standard” for NASH in pediatric patients. However, biopsy in children should be avoided, and thus it is important to assess with non-invasive diagnostic alternatives [19]. Compared to adults, in terms of histological features, steatosis was found to be significantly more severe in pediatric NAFLD cases [18].

## 4. Classic Ultrasound

### 4.1. Data in Favor of the Use of Ultrasound in Pediatric NAFLD/NASH

Classic ultrasound (US) is the most widely used imaging method for the diagnosis of fatty liver filtration due to its high availability, its user-friendliness, its tolerance by the examinees and its low cost. Typical findings are based on the ultrasonographic comparison to the right renal parenchyma, the peripheral attenuation and the presence of areas with focal amplification of the ultrasound beam. Ref. [20] The degree of infiltration can be subjectively classified as mild, moderate or severe, or as suggested by some studies, can be classified using absolute ultrasound criteria. Ref. [21,22] During a meta-analysis (2815 patients with suspected or confirmed liver disease), the exact sensitivity and specificity of the ultrasound to distinguish moderate to severe fat infiltration from no filtration, as characterized after biopsy, was 85% (80–90%) and 93% (87–97%), respectively. This study involved adult patients [23]. In children, liver US for fat detection presented a sensitivity of 70% to 85% and specificity of 50% to 60% [10,24]. Despite the diagnostic limitations for people with co-occurring kidney disease and obese patients found in the aforementioned study [23], the European directives for the management of NAFLD recommend the use of ultrasound as the first-choice imaging for people with suspected NAFLD/NASH [25].

A large pediatric cohort study showed a positive correlation between ultrasound degree of fatty infiltration and histologically established severity of the disease [26]. In the same study, a particularly interesting finding was the lack of a positive correlation between transaminase values with either ultrasound or histological findings, suggesting a lower diagnostic value of these markers in pediatric patients. Ultrasound was considered an effective tool for the staging and screening of these patients [26]. Interestingly, there has been a higher incidence of NAFLD/NASH in obese children with steatosis upon biopsy, which makes it a possible risk factor for the disease. Therefore, it is important to note that the combination of fatty liver on ultrasound with a high serum ALT value increased the detection of NAFLD in children in these highly suspected groups [27]. However, there was a remarkable percentage with conspicuous fatty infiltration on ultrasound and unaffected value of ALT in serum. In addition, it was found that children with fatty infiltration and a normal ALT value did not usually show other manifestations of metabolic syndrome. These studies rendered the ultrasound an effective tool for screening NAFLD/NASH in the suspected pediatric population, in contrast to the measurement of serum aminotransferases, which in the current literature seem to be insufficient [28]. A study in Egypt showed 100% sensitivity and 10% specificity in the detection of histologically confirmed NAFLD via ultrasound examination. Therefore, it was concluded that the absence of ultrasound or the presence of grade “A” hepatic infiltration could safely rule out histology and prevent biopsy [29].

To support the above, the use of ultrasound as a screening tool for pNAFLD/NASH showed high sensitivity but low specificity for fat infiltration in highly obese children [30]. To aid the diagnosis, MRE (magnetic resonance elastography) was proposed to detect the presence or absence of fibrosis. In an attempt to quantify the fatty infiltration by ultrasound (>5% of infiltration), there was a significant overlap with the findings of the MRE (classification: absence, mild, moderate and severe infiltration) [30]. In another group of children with histologically confirmed NAFLD, the ultrasound findings of fatty infiltration appeared to correlate well with the corresponding clinical parameters and liver pathology. In addition, the degree of hepatic fat infiltration was correlated with the fibrosis stage, and it was associated with higher BMI (body mass index), waist circumference, hematocrit and insulin resistance [31,32]. Regarding NASH, ultrasonography can provide a valid outcome, provided the use of US contrast matter, as a significant decrease has been found on the uptake of the contrast in NASH patients [33]. In addition to that, a new US fatty liver indicator score, based on the intensity of liver contrast, has been found to successfully evaluate the pathologic criteria for NASH, proving the usefulness of US for staging the disease in pediatric patients [21].

### 4.2. Non-Encouraging–Conflicting Findings

There are some practical limitations that undoubtedly reduce the diagnostic sensitivity of ultrasound in obese children, who are usually candidates for developing pNAFLD. The diagnostic sensitivity of the method is reduced either when the liver parenchyma contains percentages of fat below 30% or in people with a body mass index of 40 or more. In addition, ultrasound cannot detect the presence of steatohepatitis or fibrosis. In general, for the adult population, the sensitivity of ultrasound for the diagnosis of NASH varies from 60% to 90% with a specificity of 84% to 100%. The differences among NASH and NAFLD are not conspicuous via radiological diagnostic tools [34]. The limitations of traditional US diagnosis for pediatric patients could be eliminated by novel technologies such as controlled attenuation parameters that could detect steatosis [10].

Some opponents of the use of US argue that the low sensitivity and specificity of the method for the diagnosis and determination of fatty infiltration makes it unsuitable and inaccurate for the application of population screening for NAFLD/NASH in children. Despite the universally accepted fact that ultrasound is widely available and can rule out the presence of liver masses, cysts or bile pathology, a normal liver ultrasound cannot rule out the existence of NAFLD with absolute certainty and therefore, it is not preferred [35]. Ultrasound was also used to diagnose NAFLD with a positive prognostic value of 47% and 62%. No stable correlation was found between ultrasonic fatty infiltration and reference measurements. That does not clearly lead to the exclusion of ultrasound as a screening medium for NAFLD, but it rejects it for the staging of fat infiltration in children [36]. The same applies to the diagnosis of NASH. Several studies observed the limitations of US in NASH diagnosis, especially for obese patients and patients with hepatosteatosis less than 30% [37].

Thankfully, new technological extensions of the classic ultrasound have emerged to satisfactorily cover the need for staging of NAFLD/NASH not only in children, but also in the adult population.

### 4.3. Ultrasound and Evaluation of Therapeutic Interventions

Ultrasound seems to be used quite effectively in its classic form for the evaluation of interventions in children with NAFLD after specific periods in which a change in the metabolic profile is anticipated. A 12-month program that included diet and physical activity resulted in a significant reduction in BMI and fasting glucose, insulin, lipid and liver enzyme levels as well as hepatic ultrasound findings on conventional ultrasound [10]. Any degree of reduction in excess weight was associated with a significant reduction in the impact of NAFLD. Even a minimal reduction in excess weight led to a significant improvement in the ultrasound image, due to the reduction of fatty filtration [38].

## 5. Elastography

FibroScan, i.e., vibration-controlled transient elastography, was the first imaging method that allowed the medical community to assess the degree of hepatic fibrosis. The method evaluates a portion of the liver, representative of the total, and the hardness is expressed in kilopascals (kPa) [39]. Typically, 10 successful measurements, with an average deviation of less than 30%, are required for a reliable hardness measurement. Unfortunately, the commonly used transducer is less reliable in severely obese patients, who make up a large percentage of patients with NAFLD. For this purpose, a sound projector was constructed and has been in use for people with a BMI > 30 kg/m^2^ that penetrates deeper into the tissue of interest, overcoming the effect of thick subcutaneous fat on the final result [40]. Another important feature to consider with this method is the overestimation of liver hardness due to co-existing pathologies such as congestive heart failure, extrahepatic cholestasis and recent food intake [41].

CAP (controlled attenuation parameter) is the method by which FibroScan evaluates and quantifies liver hardness. There are numerous studies applauding its efficiency in pediatric patients. More specifically, a study of 305 overweight or obese pediatric patients participated in a CAP evaluation study. The findings were in favor of this method, as it proved to be more efficient than the classic ultrasound, which was currently used for screening children with suspected pNAFLD [42]. In another study, MRE results were used as a reference in children with NAFLD, and the CAP showed excellent diagnostic accuracy in determining the presence or absence of hepatic fatty infiltration [43]. CAP was also evaluated in children with previously documented fatty infiltrated biopsy. The method succeeded in determining the presence or absence of fatty parenchyma. CAP was also successful in the stratification of the individual degrees of infiltration as they were classified by their respective pathological reports [44]. As in NASH, CAP has been proven to be quite accurate for measuring hepatic steatosis, as well [45,46].

The acoustic radiation force impulse (ARFI) method is an alternative to the aforementioned elastography and can be performed using a standard ultrasound machine transducer. This method provides hardness measurements during a routine ultrasound examination. During the performance of a B-mode imaging, an area of interest in the hepatic parenchyma is marked for mechanical evaluation through sound waves under pressure. Hepatic hardness is expressed in m/s, and it is properly evaluated after 10 successful measurements. The two methods (CAP-ARFI) appear to have similar diagnostic accuracy; however, ARFI shows a deviation of 1.48–2.06 m/s in the diagnosis of severe hepatic fibrosis [47].

To evaluate the potential utility of ARFI for the detection of liver fibrosis in overweight and obese children with pNAFLD, a study was conducted with 148 school-age children. The results showed that children with laboratory parameters in the range of normal values, but also some with normal liver ultrasounds or with mild fatty infiltration, may in fact have had a significant degree of liver fibrosis. [48] ARFI is not only useful for the evaluation of pNAFLD in normal-weighed children, but also in obese patients or in children with ascites [49]. ARFI has shown an excellent correlation with the AST/ALT ratio in obese children and can be used as a non-invasive tool to detect pNAFLD and related hepatic parenchymal changes, especially in pediatric patients where biopsy is not a desired method [50].

The ultrasound fatty infiltration criterion is the ratio of echogenicity of the hepatic parenchyma to the renal one [51]. This ratio appeared significantly higher in a group of children with hepatic steatosis upon comparison to the corresponding index of the control group and to a group with hepatic pathologies other than pNAFLD. In addition, quantification of hepatic fat infiltration using the CAP method proved to be a safe, accurate and easily applicable method in pediatric patients [51].

ARFI diagnosis appeared to be strongly associated not only with fibrosis, but also with the level of necro-inflammatory activity. The rate of propagation of sound vibration pulses was significantly related to the degree of hepatic fibrosis in several children [52]. Marginean et al. confirmed the benefits of the performance of ultrasound elastography using ARFI technology to assess the degree of hepatic fibrosis in children with or without hepatic pathology [53]. The benefits of using this method were also reported by an extensive literature review for the non-invasive evaluation of the degree of liver fibrosis in a pediatric population with non-alcoholic liver disease [54].

These methods are also efficient in the adult population. ARFI elastography showed greater diagnostic accuracy than the accuracy obtained from visual evaluation by two experienced examiners with classical ultrasound for the initial diagnosis and evaluation of the severity of chronic fatty liver disease [55]. In the adult population, clinical consideration alongside with ARFI provides valuable, non-invasive evaluation with repetitive results; thus, it gives the green light for a new path in the management of patients with NAFLD and NASH. In general, non-invasive technics have been able to distinguish advanced liver diseases as well as NAFLD from NASH. However, fibro-genetic stage cannot be properly evaluated by radiological modalities [56].

## 6. Magnetic Elastography and MRI

Magnetic resonance elastography (MRE) is an additional non-invasive tool for diagnosing fibrosis in patients with NAFLD/NASH. The device consists of a system of active acoustic guides mounted outside a magnetic chamber. This system produces low-frequency vibrations from a guide-instrument mounted above the anatomical area of the liver [57]. There are only a handful of published studies on the efficiency of MRE in the diagnosis of NAFLD. A recent study, however, documents the superiority of MRE compared to simpler prognostic models in patients with pNAFLD confirmed by liver biopsy [58].

At the moment there is no threshold value for fat infiltration that is considered diagnostic for pNAFLD. A positive correlation was found between the degree of fatty infiltration imaging and histologically confirmed steatosis [59]. The available data concerning MRI (magnetic resonance imaging) and MRE demonstrate the growing role that these two diagnostic imaging methods are expected to play in the prevention of pediatric NAFLD/NASH. MRI-PDFF (proton density fat fraction) demonstrates a satisfactory sensitivity to changes in hepatic steatosis and combined with MRE, could be accurate enough for estimating changes in liver fat [60]. Magnetic resonance elastography is considered particularly effective in the diagnosis of pediatric NAFLD at early stages. It is also characterized as a satisfactory substitute for liver biopsy, enabling non-invasive evaluation of pNAFLD. However, it is yet unable to stage NASH due to the lack of fibrosis [46]. A liver MRI–biopsy correlation study in children revealed that the MRI contribution to the discrimination of simple steatosis from NASH in the pediatric population was not yet achieved. Even though MRI has shown an average correlation for detecting liver fat fraction in NAFLD/NASH pediatric patients, the exact progression stage of the disease is not feasible [61].

Liver biopsy, the so-far gold standard of NAFLD/NASH screening and staging, seems an inappropriate and unrealistic choice for the pediatric population. On the contrary, the increasing use of alternative, non-invasive methods, which are still under the scope of intensive research, has many potentials [62]. Transient elastography could benefit both children and adults as an alternative, non-invasive diagnostic tool for measuring hepatic stiffness and thus evaluating NASH progression [10].

## 7. Data Concerning Greece

Obesity is a major health problem, constantly growing in Greece. New data suggest that hypertension, type 2 diabetes, NAFLD/NASH and dyslipidemia have an increasing impact on the pediatric population of Greece [62].

Obesity is strongly associated with pNAFLD. In a study of 125 obese children, 75 (60%) had pNAFLD. The classic ultrasound was used to diagnose pNAFLD. Using a calibrated scale from 0 to 3, children were stratified based on the severity of fat infiltration into normal liver, mild, moderate and severe fat infiltration. The calibration was based on echo-genesis and imaging of the vascular parenchyma and septum. Liver echogenicity was measured in a longitudinal section compared to that of the adjacent right kidney [63,64]. In a study using statistical analysis of pNAFLD-related factors in children, the mechanism of homeostasis of insulin resistance as well as level of high-density lipoproteins were found to be the most significant. Insulin resistance had already been demonstrated as a factor associated with fatty liver infiltration. In addition, however, serum high-density lipoprotein levels appeared to play an additional role in the prognosis of NAFLD/NASH in obese children. In this case, the diagnosis of the disease was successfully made with a classic ultrasound [65].

## 8. Conclusions

Due to the “western” lifestyle, obesity, metabolic syndrome and, consequently, NAFLD/NASH have reached epidemic proportions in the vulnerable pediatric population around the world. This fact should greatly concern the medical community. Pediatric NAFLD is a disease which progressively develops to NASH and cirrhosis of the liver, with several intermediate stages of fibrosis. Each of these stages is a unique clinical situation that demands specific corresponding treatment. Consequently, the need for accurate diagnosis and staging of this disease is of paramount importance. Additionally, screening methods in risk populations such as children with non-intervention tools are essential in order to balance the risk–benefit ratio. In this context, the current literature considers the classic ultrasound as the most suitable screening method and the first-choice diagnostic tool. This non-interventional tool applies to adults, as well, in order to diagnose and stage the disease. Elastography and ARFI are more suitable for determining the stage of hepatic fibrosis. Since the new classic ultrasound machines incorporate the ARFI method, we conclude that ultrasound technology, being thoroughly studied and widely available, is the proper tool for the medical specialists who track, monitor and treat pediatric NAFLD/NASH. MRE has shown great potential as a diagnostic non-invasive tool that could successfully stage fibrosis and hepatic inflammation. New evidence will continue to support the international literature and validate the conclusion of this study.

## Data Availability

Not applicable.
